# Improved anti-cancer effect of epidermal growth factor-gold nanoparticle conjugates by protein orientation through site-specific mutagenesis

**DOI:** 10.1080/14686996.2021.1944783

**Published:** 2021-09-06

**Authors:** Aiwen Zhang, Jun Nakanishi

**Affiliations:** a Graduate School of Advanced Science and Engineering, Waseda University, Shinjuku-ku, Tokyo, Japan; b Research Center for Functional Materials, National Institute for Materials Science (NIMS), Tsukuba, Japan

**Keywords:** Nanomedicine, EGF-nanoparticle conjugates, protein orientation, phosphorylation signaling, growth inhibition, binding activity, 30 Bio-inspired and biomedical materials, 211 Scaffold / Tissue engineering/Drug delivery, 212 Surface and interfaces, Nanoparticles

## Abstract

Epidermal growth factor (EGF)-nanoparticle conjugates have the potential for cancer therapeutics due to the unique cytotoxic activity in cancer cells with EGF receptor (EGFR) overexpression. To gain its maximum activity, the EGF molecule should be immobilized on the nanoparticle surface in a defined orientation so as the bulky nanoparticle will not interfere EGF-EGFR interaction. Herein, we demonstrate successful enhancement of the anti-cancer activity of EGF-gold nanoparticle conjugates (EGF-GNPs) by controlling the EGF orientation on the surface of the nanoparticle through site-specific mutagenesis. Three lysine-free EGF variants (RR, RS, and SR) were designed, where two endogenous lysine residues were replaced with either arginine (R) or serine (S). The EGF mutants can be conjugated to the GNPs in a controlled orientation through the single amino group at the N-terminus. The ability of the mutants to induce extracellular signal-regulated kinase (ERK) phosphorylation was no different from wild type EGF (WT) in soluble form, rather lowered for one mutant (RR). However, after conjugated to GNPs, the SR mutants exhibited an enhanced biological activity than WT, in terms of ERK phosphorylation and growth inhibition of cancer cells. Further analysis of the binding constant of each mutant indicated the emergent enhanced activity of the GNP conjugates of the SR mutant was not solely contributed to the orientation, but to its higher binding activity to EGFR. These results validate the present genetic recombination strategy to improve the anticancer efficiency of EGF-GNPs.

## Graphical abstract

**Figure:**
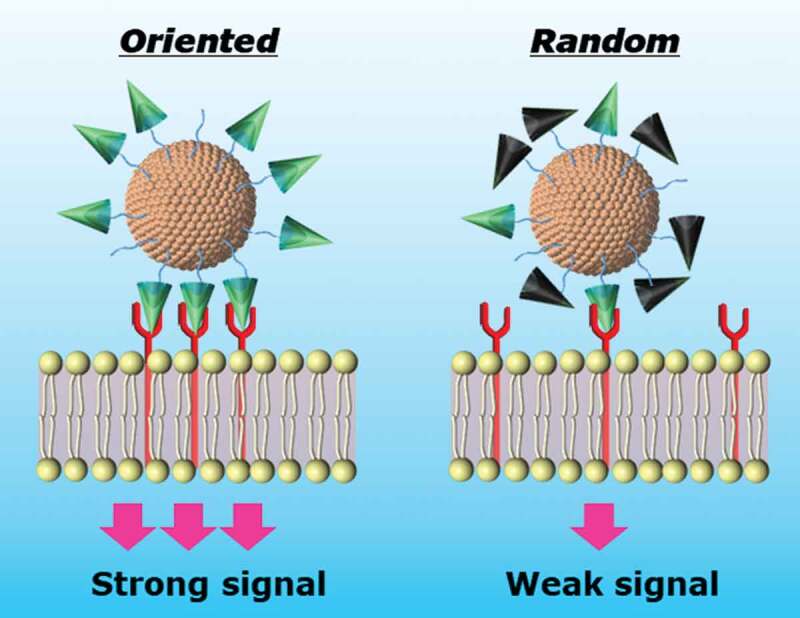

## 1. Introduction

Nowadays, cancer is still one of the leading causes of death in the world [1]. Treatment of cancer by chemotherapy, surgery, and radiation therapy has been proven to be effective in treating many cells. However, most of them are accompanied with harmful side effects to normal cells, therefore the development of new therapeutic methods is urgently needed [2–4]. The nanomedicine, including nanoparticle-based and two-dimensional (2D) nanoarchitecture-based vehicles, which carrying chemical drugs or protein/peptide drugs are promising to be the new generation medicines due to the improved capacity of the drug-loading, efficacy of cell uptake, and biocompatibility [5–8]. However, the specify of them still needs to be enhanced to selectively kill tumor cells and reduce cytotoxic effects to the normal cells. Recently, the epidermal growth factor receptor (EGFR)-targeted cancer therapeutics has been developed due to the overexpression of EGFR in many tumors [9]. The EGFR is the membrane receptor and plays an important role in cell growth and proliferation [10]. Abnormally activated EGFR by receptor overexpression, mutation, and ligand-independent activation can result in the development of cancer [11]. Therefore, specific EGFR inhibition is one of the promising methods for cancer therapy. There are two major approaches of EGFR inhibition with different mechanism by using monoclonal antibodies (mAbs) and tyrosine kinase inhibitors (TKIs). The mAbs (cetuximab, panitumumab) against EGFR are designed specifically to recognize extracellular domain of EGFR to compete with endogenous ligands of EGFR, leading to the inhibition of activation of the EGFR tyrosine kinase induced by the ligand [11–14]. The TKIs such as gefitinib, erlotinib, and canertinib, are small molecules which bind to the intracellular catalytic domain of EGFR tyrosine kinase to compete with adenosine-5ʹ-triphosphate (ATP), inhibiting autophosphorylation and activation of downstream signaling [11,12,15]. Both strategies of inhibition of EGFR have been approved for clinical use and have effective anti-cancer activity. In spite of the effective treatment, it is still difficult to completely cure patients due to the developed resistance of tumor to the EGFR inhibitors. Therefore, the development of new anti-EGFR drugs based on different approaches are expected. Recently, it has been identified that the conjugation of epidermal growth factor (EGF) to gold nanoparticles (GNPs) can gain an enhanced apoptotic efficiency in cancer cells [16,17]. Our group has identified that the emergent unique apoptosis activities of the EGF-GNP conjugates were attributed to the confinements of EGFR within lipid rafts and selective activation of extracellular signal-regulated kinase (ERK) [18]. The EGF conjugates is promising due to the alternative apoptosis pathway of EGFR to overcome the limitation of resistance of inhibitors, and it allows precise delivery of the therapeutics to the intended cell targets which overexpressed EGFR.

**Figure 1.**
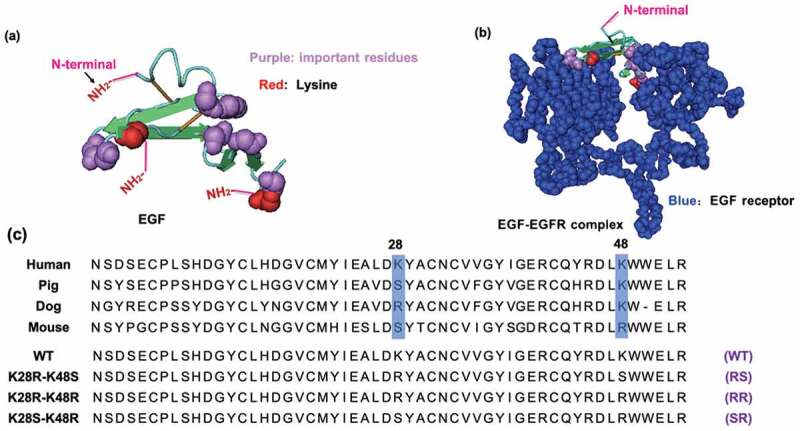
Three-dimensional structures of human EGF (PDB: 1JL9) and EGF/EGFR complex (PDB: 1IVO), and the amino acid sequences of wild type (WT) and mutant EGF. (a) The 3D structure of human EGF. Red and purple colors represent lysine residues and important residues, respectively. (b) The structure of EGF-EGFR complex. The EGFR is shown in the space-fill model with blue color. (c) Sequential alignments of EGF from various species and designed mutant EGF. The residues 28 and 48 corresponding to two lysines in human EGF are highlighted by blue boxes. Two lysine residues of WT EGF were replaced with either arginine or serine in the designed mutants EGF

Given the importance of EGF-conjugates in cancer treatment, it is crucial to improve the capability of EGF-conjugates to inhibit cancer cell growth and increase anti-cancer effect with its low dosage. According to the reported work [16–20], the commonly used methods for preparing of EGF conjugates is to react the EGF molecule with nanoparticles by chemical coupling using primary amino groups of EGF. However, there are three amino groups in the human EGF molecules, *i.e*. N- terminus, Lys28, and Lys48, that can cause the diversity of the coupling reaction. Moreover, based on its crystal structure (Figure 1a), all these sites are not buried inside the protein and located at the distinct positions of the EGF molecule, thereby there are three possible orientations of the immobilized EGF on the surface of GNPs. Especially, it should be noted that the Lys28 and Lys48 residues are located close to the interfaces where the EGF molecule bind to EGFR (Figure 1b). This means if EGF is conjugated to the GNPs through these lysine sites, its binding to EGFR could be interrupted by the presence of bulky GNPs.

Given the foregoing, we hypothesized that it would become more favorable for the EGF-GNP conjugates to interact with EGFR if we can limit the conjugation position to the N-terminus of EGF. Herein, we designed and prepared three modified human EGF, containing mutations of the lysine (K) either to arginine (R) or serine (S) using gene recombination technology, to produce three lysine-free EGF (RR, RS, SR) (Figure 1c). The bioactivity of these mutant EGF were confirmed. Following, the EGF mutants were conjugated to GNPs at their N-terminus. The bioactivity of the mutant EGF-conjugates in terms of ERK activation and growth inhibition of cancer cells were also determined to verify the present strategy to improve anti-cancer effect of EGF-GNP conjugates.

## 2. Materials and methods

### 2.1 Materials

The pET41a, biotinylated thrombin, and streptavidin-agarose resins were obtained from Merck (Darmstadt, Germany). The Origami™ 2 (DE3) Singles™ Competent Cells, BugBuster protein extraction reagent, and benzonase were purchased from EMD Millipore (Burlington, Massachusetts, USA). The kanamycin, isopropyl-ß-d-thiogalactopyranoside (IPTG), triethylamine, dimethyl sulfoxide (DMSO), penicillin-streptomycin solution, and bovine serum albumin (BSA) were obtained from Wako (Osaka, Japan). The glutathione sepharose 4B beads was obtained from GE-healthcare (Chicago, Illinois, USA). The anti-EGF rabbit antibody was purchased from Abcam (Cambridge, UK). The phospho-ERK1/2 (T202/Y204) rabbit monoclonal antibody was obtained from Cell Signaling Technology (Danvers, Massachusetts, USA). The dithiobis (succinimidyl undecanoate) (DSU) and 2-(*N*-morpholin)ethanesulfonic acid (MES) was obtained from Dojindo (Kumamoto, Japan). The ω-methoxy-poly(ethylene glycol) amine (mPEG-NH_2_, Mw = 5000) was obtained from NOF Corporation (Tokyo, Japan). Fifteen nm gold nanoparticle was purchased from BBI Solutions (Krumlin, UK). Fetal bovine serum (FBS) was obtained from BioWest (Nuaille, France). Cell proliferation assay kit (CellTiter 96®Aqueous Non-radioactive Cell Proliferation) was purchased from Promega (Madison, Wisconsin, USA). The 3,3ʹ,5,5ʹ-tetramethylbenzidine (TMB) was obtained from KPL (Gaithersburg, Maryland, USA). The Micro BCA™ Protein Assay Kit and *N*-hydroxy succinimide (NHS) were obtain from Thermo Fisher Scientific (Waltham, Massachusetts, USA). Dulbecco’s Modified Eagle’s Medium (DMEM), anti-rabbit IgG horseradish peroxidase (HRP), 1-ethyl-3-(3-dimethylaminopropyl)carbodiimide (EDC), poly(ethylene glycol) 2-mercaptoethyl ether acetic acid (SH-PEG-COOH), anti-rabbit IgG alkaline phosphatase, 4-nitrophenyl phosphate disodium salt hexahydrate, and triton X-100 were obtained from Sigma-Aldrich (St. Louis, Missouri, USA). The primers: P1, P2, P3, P4, and P5 were also prepared by Sigma-Aldrich.

### 2.2 Cloning of mutant EGF without lysine site

The cDNA sequences of the wild type (WT) and mutant hEGF were prepared by polymerase chain reaction (PCR) by using corresponding primers listed in Table 1. First, cDNA of WT EGF was amplified by PCR using hEGF-His_6_ [21] as a template and a pair of primers P1 and P2. Then, the reverse sequence of the PCR product was used as a mega-primer, and together with the T7 promoter, the cDNA of glutathione-S-transferase (GST)-tagged WT EGF sequence was again amplified by PCR and subcloned into the XbaI/NotI site of pET-41a. The pET-41a vector contains cDNA sequence of thrombin cleavage site between the GST tag and multi-cloning sites. From the WT EGF, the cDNA of mutant EGF (RR, RS, SR) were further produced by the PCR using the following pairs of primers, RR: P1 and P3; RS: P1 and P4; SR: P1 and P5.

**Table 1.** The sequences of primers used for the preparation of EGF mutants. The codons corresponding to the mutation sites are shown in lower-case letters

P1	5’	-	GCGGGTCTGGTGCCACGCGGATCCAATAGTGACTCTGAATGTCC
P2	5’	-	CCCGCGGCCGCTAGCGCAGTTCCCACCACTT
P3	5’	-	CCCGCGGCCGCTAGCGCAGTTCCCACCAtctCAGGTCTCGGTACTGA
			CATCGCTCCCCGATGTAGCCAACAACACAGTTGCATGCATAtctGTC
			CAATGCTTCAATATAC
P4	5’	-	CCCGCGGCCGCTAGCGCAGTTCCCACCAggaCAGGTCTCGGTACTGA
			CATCGCTCCCCGATGTAGCCAACAACACAGTTGCATGCATAtctGTC
			CAATGCTTCAATATAC
P5	5’	-	CCCGCGGCCGCTAGCGCAGTTCCCACCATCTCAGGtctCGGTACTGA
			CATCGCTCCCCGATGTAGCCAACAACACAGTTGCATGCATAggaGTC
			CAATGCTTCAATATACAT

### 2.3 Expression and purification of mutant hEGF

The pET-41a vector containing the fusion construct GST-hEGF was transformed into *Escherichia coli* (*E. coli*) Origami 2 (DE3). The transformed cells were inoculated in LB culture medium containing kanamycin at 37°C overnight. On the next day, the overnight culture was used to inoculate and grow in 500 mL LB medium containing kanamycin at 37°C on a shaker. At an optical density of A_600_ = 0.6, the IPTG was added to induce protein expression. Subsequently, the harvested and pelleted cells were lysed in BugBuster protein extraction reagent containing benzonase. The supernatant was transferred to PBS-equilibrated glutathione sepharose beads. Then, the mixture of the lysate and GS beads were transferred to the column and washed with ice-cold thrombin-digestion buffer. Afterwards, the GS beads with thrombin-digestion buffer were transferred to a tube. The biotinylated thrombin was added. After overnight reaction, the mixture was centrifuged. The supernatant was collected and incubated with avidin-agarose resins at 4°C for 30 min. Finally, the supernatant was centrifuged briefly and transferred to a fresh tube. The extracted WT and mutant EGF were analyzed by sodium dodecyl sulfate polyacrylamide electrophoresis (SDS-PAGE) by using Any kD™ Mini-Protein® TGX ™ gels (Bio-rad). The protein concentration of the hEGF was quantified by direct enzyme-linked immunosorbent assay (ELISA) method which described in the earlier paper [18]. Briefly, after overnight coating, the EGF coated 96 well-plate was washed and blocked with 1% BSA for 1 h, and then incubated with rabbit anti-human EGF antibody followed by anti-rabbit IgG horseradish peroxidase (HRP). The signal was detected after adding TMB substrate by micro-plate reader (Bio-Rad, Hercules, California, USA) at 450 nm.

### 2.4 GNPs PEGylation and functionalization with mutant EGF

We synthesized the disulfide molecule bearing poly(ethylene glycol) (PEG-DSU) according to the procedure reported in the previous paper [22]. Two hundred fifty µL of 1.25 mM of DSU and 500 µL of 2.5 mM mPEG-NH_2_ were mixed with 250 µL of 2.5 mM triethylamine in DMSO, and the mixed solution was reacted overnight by using a rotating mixer at 8 rpm at room temperature. Six mL of 15 nm GNPs was concentrated by centrifugation at 10,000 rpm for 30 min at room temperature into a 60 µL solution. Then, the GNPs were functionalized in a DMSO solution containing 2 mM PEG-DSU and 2 mM SH-PEG-COOH using a rotating mixer at 8 rpm at room temperature overnight. After an overnight reaction, the functionalized GNPs were washed with Milli-Q water by centrifugation (12,000 rpm, 30 min, three times) and suspended by 600 µL MES buffer (pH = 5). Subsequently, 150 µL of 0.2 M EDC and 150 µL of 0.2 M NHS were added and placed on a rocker for 20 min at room temperature. After reaction, the mixture was washed by centrifugation (12,000 rpm, 30 min, three times) and suspended in PBS containing 2 μM EGF. The mixture was placed on a rocker for 2 h at room temperature. Afterwards, the EGF conjugated GNPs were washed by centrifugation (12,000 rpm, 30 min, three times). Subsequently, 400 µL of 50 mM Tris (PH = 7.6) was added and mix overnight. On the next day, the mixture was washed by PBS and resuspended in medium for further using. The number of EGF on a single GNP was determined using the following formula: number of EGF on one GNP = (total EGF added-remained EGF in the supernatant)/total concentration of the GNPs. The amount of EGF in the supernatant was calculated by the Mirco-BCA assay according to manufacturer’s introduction by using the commercial EGF as standard. The concentration of GNPs was detected by UV-Vis spectrophotometer.

### 2.5 Characterization of EGF-GNPs

The dynamic light scattering (DLS, DLS-8000HAL, Otsuka Electronics, Osaka, Japan) was used to evaluate the size distribution of EGF-GNPs during functionalization at a scatting angle of 90°C under 488 nm laser. The UV-Vis spectrum was obtained by a UV-Vis spectrophotometer (UV-2600, Shimadzu, Kyoto, Japan).

### 2.6 Cell culture

A431 cells (RCB0202) were obtained from the RIKEN cell bank (Ibaraki, Japan) and cultured in DMEM supplemented with 10% FBS and 1% penicillin-streptomycin at 37°C in a humidified atmosphere containing 5% CO_2_.

### 2.7 EGF Cell binding assay

To measure the binding affinity of mutants EGF to EGFR, A431 cell were seeded at a density of 20,000 cells/well in 96-well plates overnight. On the next day, cells were starved for 4 h followed by treating with various concentrations of mutants EGF. Then, cells were fixed in 4% paraformaldehyde for 15 min at room temperature and washed three times by PBS. Subsequently, the wells were blocked by incubating with 1% BSA in PBS for 1 h at room temperature. Subsequently, the wells were washed with PBST (0.05% Tween-20 in PBS) followed by adding 100 µL of anti-EGF antibody (1:1000) for 2 h at room temperature. After washing again with PBST, 100 µL of anti-rabbit IgG horseradish peroxidase (HRP) (1:10,000) was added for 45 min at room temperature. Subsequently, the wells were washed and 100 µL TMB solution as a substrate was added to each well to detect the bound HPR. After 30 min, the reaction was stopped by an addition of 100 µL of 1.0 N hydrogen chloride solution and the signal was recorded at 450 nm by the microplate reader (Model 6800, Bio-rad, Hercules, California, USA).

### 2.8 ERK activity assay by in situ cell ELISA

A431 cells were seeded into a 96-well plate at a density of 20,000 cells/well overnight. Following, the medium was substituted with 50 µL medium without FBS to serum-starve cells for 4 h. Cells were treated with various EGF-GNPs, free EGF, or DMEM for 5 min at 37°C. Afterwards, the medium was removed, and the cells were washed by PBS followed by adding 150 µL of 4% paraformaldehyde in PBS for 20 min at room temperature. After washing with PBS three times, the cells were permeabilized with 150 µL of 0.5% Triton X-100 in PBS for 20 min. Subsequently, the cells were blocked with 2% BSA in PBS for 1 h. Then, 50 µL phospho-ERK1/2 (T202/Y204) rabbit monoclonal antibodies (1:1000) was added to each well for 2 h. After another round of washing, 100 µL of anti-rabbit IgG alkaline phosphatase (1:3000) was added. Subsequently, the cells were washed again followed by adding 100 µL 4-nitrophenyl phosphate disodium salt hexahydrate as a substrate. The signal was recorded by the microplate reader at 405 nm.

### 2.9 Cell viability assay

A431 cells were cultured in a 96-well plate (4000 cells/well). After overnight, the cells were treated with EGF-GNPs, soluble EGF, or DMEM for 72 h. Then, the medium of cells was gently removed and washed twice by PBS. Afterwards, 100 µL medium was added to the well. The cell viability was measured by cell proliferation assay according to the manufacturer’s protocol. After 2 h incubation, the absorption was measured by the microplate reader at 490 nm.

### 2.10 Data analysis

The variances of the results were determined by a two-tailed *F*-test with a null hypothesis of equal variance. Following a two-tailed student’s *t*-test was used to determine the statistically significant of the data of each group. The *P*-value lower then 0.05 was interpreted as statistically significant. The GraphPad Prism 9.0 software was used to calculate the equilibrium dissociation constant values (K_d_) by nonlinear regression curve following applied one site binding (hyperbola).

## 3. Results and discussion

### 3.1 Molecular design of EGF mutants

**Figure 2.**
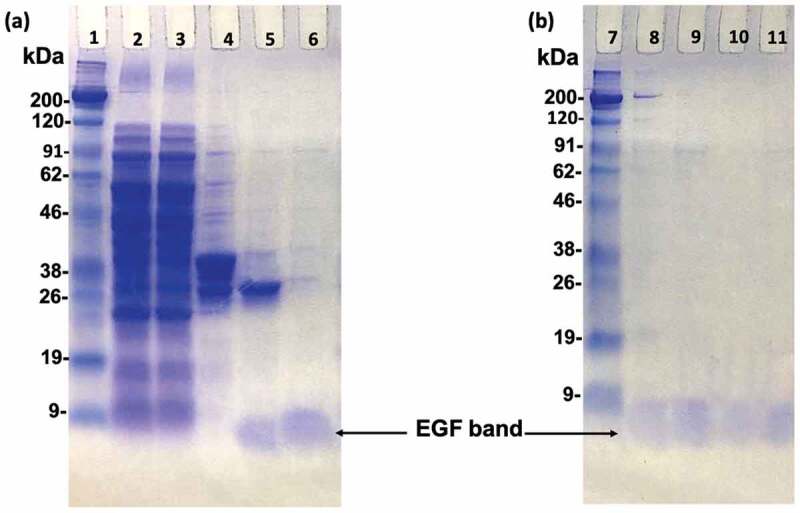
SDS-PAGE analysis of EGF variants during purification. Polyacrylamide gels were stained with Coomassie Brilliant Blue. Lane 2; total soluble protein from the supernatant of the cell lysate. Lane 3; supernatant that did not bound to the GS resin. Lane 4; the adsorbate of the GS resin eluted by SDS-loading buffer. Lane 5; the eluted proteins after thrombin digestion. Lane 6; wild type EGF after biotin-avidin purification. Lane 8, 9, 10, and 11; comparisons of WT, SR, RS, and RR EGF after final biotin-avidin purification. Lane 1 and 7; protein markers. The EGF bands are indicated by arrows

In order to prepare the oriented EGF, the lysine of EGF needs to be mutated to other amino acid residues without primary amine group, so that we can obtain EGF mutants with a single primary amine group at the N-terminal amine group. Previous study applied phage-displayed libraries of EGF to select EGF variants without lysine and revealed a diverse set of residues was found at K28 and K48 [23]. This means these two residues are capable of replacing with other residues without losing binding ability to EGFR. More specifically, one EGF variant (K28Q, R45S, K48S, and R53S) showed an identical binding activity to the WT EGF. Therefore, the K48S mutation seemed not interfere with the binding affinity of EGF. In addition, Campion et al. reported a slight increase in binding affinity of the K28R mutant to the EGFR [24]. Combining these, we designed our own EGF mutants (RR, RS). Besides, we utilized homologous residues from the EGF sequenced of various species and designed another mutant EGF (SR), which the amino acids at the sites 28 and 48 were replaced with serine (S) and arginine (R), respectively (Figure 1c). We then used the genetic recombination technology to prepare the mutant EGF. After the PCR reaction, the produced plasmid DNA encoding EGF with single NH_2_ group was inserted into a pET-41a vector containing the GST-thrombin tag sequence. Then, the plasmids were transformed in *E. coli* Origami 2 (DE3) and various EGF mutants were purified from bacterial pellet. SDS-PAGE analysis confirmed that the various EGF mutants were successfully prepared with correct size of 6 kDa after digestion of the GST residue with biotinylated thrombin and the purification through the biotin-avidin reaction (Figure 2). As shown in Figure 2, the lane 8, 9, 10, and 11 were the WT, SR, RS, and RR bands after purification, respectively. No other bands except the EGF band in these lanes can be observed, revealing the high purity of the prepared protein.

### 3.2 Bioactivity of mutant EGF

**Figure 3.**
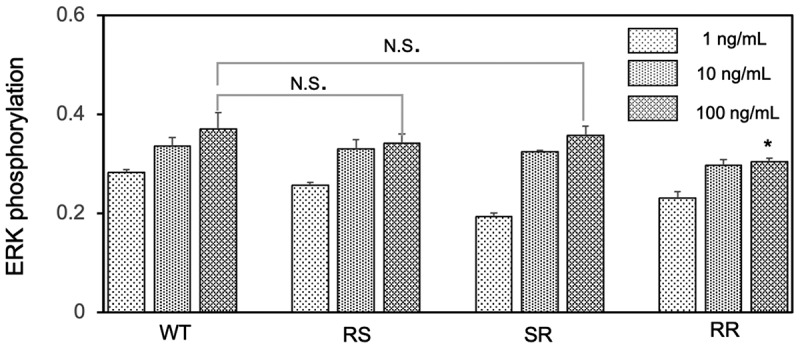
Comparison of the activity of EGF variants in the soluble form. A431 cells were treated with each EGF variant at concentration of 1 ng, 10 ng and 100 ng/mL for 5 min and the ERK phosphorylation levels were evaluated by in situ cell ELISA. The asterisk in the RR group means represents significant difference against WT at the concentration of 100 ng/mL

Herein, we used ELISA to evaluate the biological activity of EGF mutant from the phosphorylation of ERK in EGFR-overexpression A431 cells. The ERK is an essential signal transduction protein that transmits mitogen signals to intracellular targets and can be activated by the EGF binding to the EGFR, leading to the phosphorylation of ERK [25]. The abundance of phosphorylated ERK in the cell sample serves as an indicator of the level of bioactivity of mutant EGF [26]. Figure 3 shows the changes in the relative ERK phosphorylation levels after treating the cells with different concentrations of mutant EGF and WT EGF. The level of ERK phosphorylation treated with the EGF mutants and WT show dose-dependent increases with the concentration increasing. The ERK phosphorylation did not appear to increase obviously between the 10 ng/ml and 100 ng/ml groups presumably because of the reaction closing to the saturation. The similar saturation of ERK phosphorylation in the higher EGF concentration was also observed in our previous paper [18]. When focusing on the maximum responses, two mutants (RS, SR) have the comparable activity as WT EGF in the soluble form, while the RR mutant shows lower activity. The result of the low bioactivity for the RR mutant is consistent with the study described by Bachran et al, where the EGF variant (RR) fused to a toxin showed decreased enzymatic activity [27]. Based on this, we only used the RS and SR mutants to further study of the conjugates with the GNPs.

### 3.3 Preparation and characterization of EGF-GNPs conjugates

**Figure 4.**
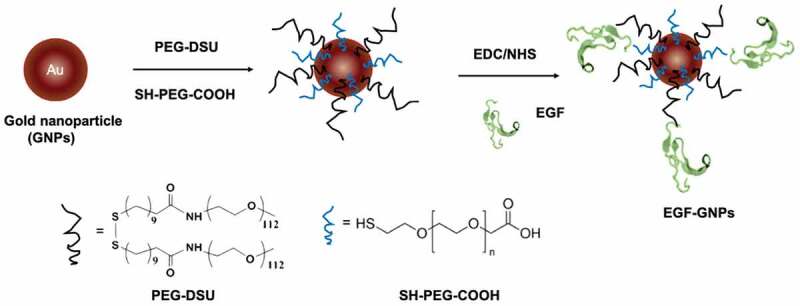
The schematic diagram of the procedure for EGF-GNPs conjugates preparation

**Figure 5.**
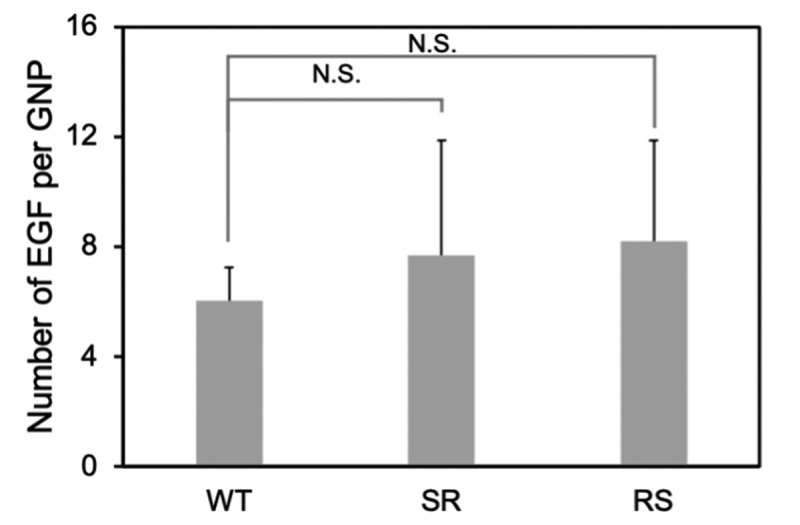
Number of EGF molecules conjugated to single nanoparticle. The average number of EGF per single GNP was calculated based on the decrease in the EGF concentration in the supernatant and the concentration of GNP

**Figure 6.**
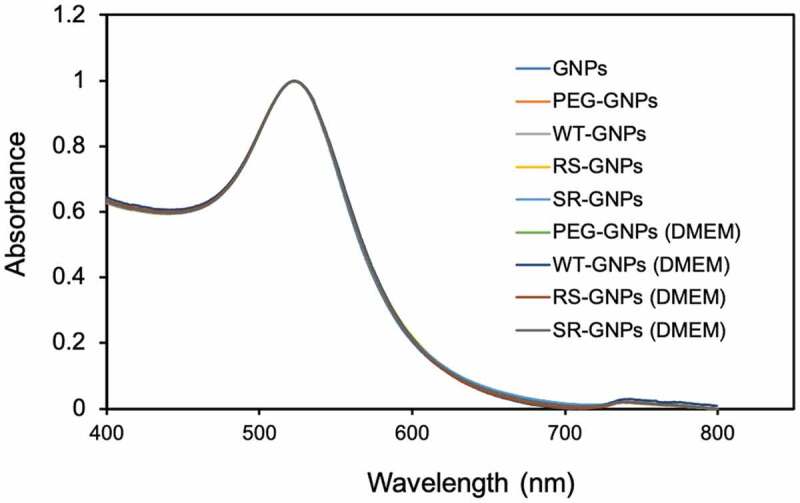
UV-Vis spectra: The initial GNPs (GNPs) in water, the functionalized GNPs without EGF (PEG-GNPs) and with various EGF-GNPs in the buffer (0.1X PBS). The data for PEG-GNPs and various EGF-GNPs after dispersion in the serum-containing cell culture medium for 1 day are shown, which are labeled as PEG-GNPs (DMEM), WT-GNPs (DMEM), RS-GNPs (DMEM) and SR-GNPs (DMEM)

Two different polyethylene glycol (PEG) molecules, HS-PEG-COOH and PEG-DSU, were used to functionalize the NP surface. The PEG-DSU introduces PEG brushes with molecular weight around 5000, which has been adopted to increase the stability of nanoparticles and reduce non-specific adsorption of proteins. The HS-PEG-COOH was used to covalently bind EGF at its terminal carboxy group. The procedure of functionalization of EGF-GNPs was shown in Figure 4. Firstly, 15 nm GNPs were functionalized with HS-PEG-COOH and PEG-DSU. After functionalization, the GNPs were reacted with EDC/NHS to activate the carboxyl group of HS-PEG-COOH. Following, the activated GNPs were reacted with various EGF (WT, RS, SR). The average number of mutant EGF molecules conjugated to a single nanoparticle was within the range of 6–8 (Figure 5). The number of EGF (WT, RS, SR) shows no significant difference between each other. Meanwhile, the stability of the GNP conjugates has been checked by the UV-Vis spectroscopy (Figure 6) and DLS (Table 2). The increased diameter changes of PEG-GNPs and EGF-GNPs compared with the GNPs (initial GNPs) confirmed the successful functionalization of GNPs (Table 2, before). Considering the functionalized GNPs will be later used in the cell culture medium with serum, which may induce the GNPs aggregation, we also studied the stability at that condition. The measured UV-Vis spectra (Figure 6) of EGF-GNPs dispersed in the culture medium were almost perfectly superimposed with those of EGF-GNPs dispersed in the 0.1X PBS. These DLS and UV-Vis results demonstrated the successful chemical modification and the stability of EGF-GNPs under physiological conditions.

**Table 2.** The hydrodynamic diameter and polydispersity index (PDI) of the functionalized GNPs before and after dispersion in the serum-containing cell culture medium

	Before		After	
	Size (nm)^‡^	PDI	Size (nm)	PDI
GNPs^†^	13 ± 2	0.06	–	–
PEG-GNPs^†^	25 ± 5	0.07	24 ± 5	0.14
WT-GNPs	29 ± 6	0.17	29 ± 6	0.20
SR-GNPs	28 ± 6	0.19	28 ± 6	0.19
RS-GNPs	28 ± 6	0.18	28 ± 6	0.21

^†^GNPs: Commercial GNPs; PEG-GNPs: non-EGF-functionalized GNPs.

^‡^Determined by DLS measurements and averaged based on number distribution.

### 3.4 Bioactivity of EGF-GNPs

**Figure 7.**
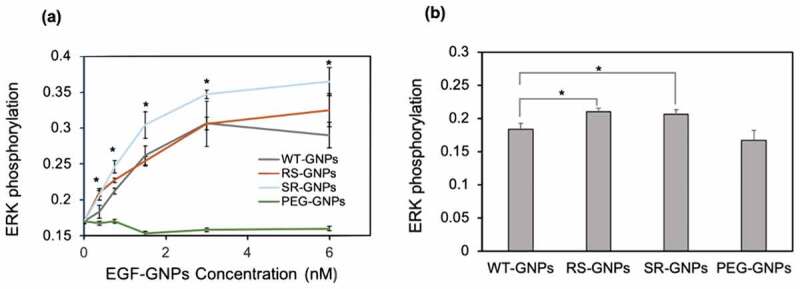
Comparison of the activity of EGF variants in the GNP conjugates. ERK phosphorylation levels were detected by in situ cell ELISA in A431 cells treated with EGF-GNPs and non-EGF-functionalized GNPs (PEG-GNPs) for 5 min. (a) The dose dependent curves of ERK phosphorylation. (b) Bar graph representations of the ERK phosphorylation intensities data at 0.38 nM nanoparticles, extracted from a. Each point was mean ± SD from three independent experiments (*P < 0.05, **P < 0.01)

Same as soluble EGF, we evaluated the bioactivity of the mutant EGF-GNPs by using ERK phosphorylation assay [26]. Figure 7a shows the relative phosphorylation levels of ERK in A431 cells in response to EGF conjugates (WT-GNPs, SR-GNPs, and RS-GNPs) at various concentrations. The ERK phosphorylation level of A431 was elevated with the concentration of the EGF-GNPs conjugates increasing. Whereas the control GNPs with no EGF conjugation did not show such increase in the phosphorylation level. These results confirm that both mutants (RS and SR), as well as WT, retain biological activity even after conjugation to GNPs. When comparing the ERK phosphorylation levels among the EGF variants, the SR conjugates show significantly higher levels than WT conjugates throughout all particle concentrations. On the other hand, the RS conjugates showed slightly increased phosphorylation level than WT, but significant differences were only observed in the lower particle concentrations (0.38 nM and 0.75 nM; Figure 7b depicts the difference at 0.38 nM). It should be noted that the SR mutant showed significantly higher bioactivity than WT in the conjugates form, while it showed almost identical bioactivity to WT in the soluble form at 100 ng/mL and even lower than WT at 1 ng/mL. This means the controlled orientation of SR on the surface of the GNP enhanced the efficiency to interact with EGFR leading to an increased activation of downstream signal at ERK. On the other hand, limited enhancement in the ERK phosphorylation for the RS mutants indicate the effect of the orientation control of the RS mutant on the activity enhancement of the nanoparticle conjugates is somehow moderate.

### 3.5 A431 cell growth inhibition after treating with EGF-GNPs

**Figure 8.**
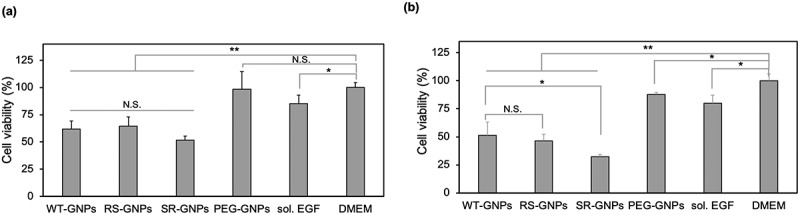
Comparison of growth inhibitory activity of EGF variants in the GNP conjugates. A431 cells were treated either with the GNP conjugates of EGF variants, non-EGF-functionalized GNP (PEG-GNPs), soluble EGF (sol. EGF), and medium for 3 days. Cell viability was calculated based on the growth inhibitory effects relative to those treated with DMEM. The results of nanoparticle concentration at (a) 6 nM and (b) 12 nM are shown. The concentration of soluble EGF were set at 48 nM and 96 nM, respectively, to keep the effective EGF concentration identical to EGF-GNPs. Each data represents mean ± SD from three independent experiments (*p < 0.05, **p < 0.01)

To monitor the effect on cell growth, A431 cells were incubated with the different concentrations of the mutant EGF-GNPs, WT-GNPs, PEG-GNPs, and DMEM (as a buffer). The effect of free EGF was also evaluated as a positive control based on its well-known growth inhibitory effects in EGFR-overexpressing cancer cells. In Figure 8a, we showed the cell proliferation of A431 cells treated with WT and mutant EGF-GNPs, or PEG-GNPs at 6 nM. The concentration of soluble EGF was set at 48 nM to keep the effective EGF concentration identical to those of EGF-GNPs (6 nM x 8 molecule/GNP). The multiplier 8 here was the maximum value of the number of the EGF molecules conjugated to the single GNP (6–8) for WT and mutant EGF based on the above calculation. The results show both EGF-GNPs and free EGF can result in a significant reduction of the cell proliferation. In addition, the proliferation in the presence of PEG-GNPs at the same 6 nM concentration shows not much difference with control (only DMEM treatment). We conclude that the influence of GNPs themselves to the growth of A431 cells is negligible under the chosen experimental conditions. Also, the cell growth inhibition by EGF-GNPs was significantly lower than the free EGF even though their effective concentrations were identical. This fact demonstrates the enhanced growth inhibitory effect of EGF-GNPs in comparison with free EGF. When comparing the cell proliferation in the presence of SR-GNPs, RS-GNPs, and WT-GNPs, there was no significant difference between each other, showing the identical growth inhibition to the cancer cells. However, we could see slightly lower viability for SR-GNPs. Therefore, we further increased the nanoparticle concentration up to 12 nM (Figure 8b). Even though, in this high concentration, the additions of GNPs showed slight reduction in cell viability in general, we could see a significantly enhanced cytotoxicity for the SR-GNPs treatment compared to those treated with WT-GNPs. Besides, the viability of the cells treated with RS-GNPs show no significant difference with cells treated with WT-GNPs. According to these data, the SR-GNPs demonstrate the highest growth inhibition to the A431 cells compared with other mutants EGF-GNPs and WT-GNPs. The results agreed with the ERK phosphorylation results, indicating possible relationship between ERK phosphorylation and the cell growth inhibition.

### 3.6 The binding affinity of mutant EGF for EGFR

**Figure 9.**
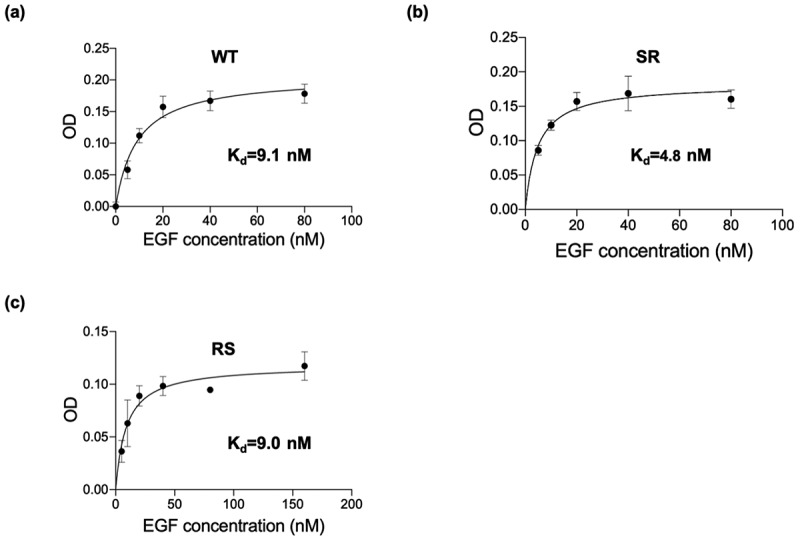
Comparative assessment of the receptor binding affinities of mutants EGF and WT EGF. A431 cells were treated with soluble EGF variants at given concentrations for 4 hours and the amount of bound EGF was detected by in situ cell ELISA using anti-human EGF antibodies based on the procedures described in the experimental section. Each value is mean ± SD from three independent experiments. The K_d_ values were calculated by nonlinear regression by using GraphPad Prism 9 software

To investigate the reason for the enhanced activity of the SR mutant compared to WT, we analyzed the binding affinity of various EGF (RS, SR) and WT-EGF for EGFR expressed in A431 cells by means of in situ cell ELISA [28]. The equilibrium dissociation constant values (K_d_) were evaluated using a nonlinear regression analysis. As shown in Figure 9, all EGF variants bound to EGFR in a concentration-dependent manner and showed a typical receptor-ligand binding mode. Under this assay condition, the estimated K_d_ value of WT was 9.1 nM. Meanwhile, the estimated K_d_ value for SR and RS was 4.8 nM and 9.0 nM, respectively. The K_d_ value revealed a 1.9-fold lower for SR compared with WT. Early study demonstrated decreased cytotoxicity for an EGF mutant with a lower binding affinity to EGFR in MDA-MB-468 (overexpressed EGFR) [28], therefore the increased affinity of the SR mutant toward EGFR is supposed to be involved in its increased growth inhibitory effect.

## 4. Discussion

Emergent cytotoxic activity of EGF in its nanoparticle conjugates is considered as an alternative cancer therapeutics, which conventional EGFR inhibitors have been hampered by the development of drug resistance in some tumor types. This study intended to improve the anti-tumor efficacy of EGF-GNPs by anticipating the reduced activity of the conjugated EGF molecule on the nanoparticle surface in early work. This concern is derived from the possible diversity of the coupling reactions between the EGF molecule and GNPs due to the existence of three primary amines, *i.e*. N-terminus, Lys28, and Lys48, in endogenous human EGF molecule. Herda et al. showed the antibodies grafted on the nanoparticle surface had low probability (about 4%) to have a suitable orientation for recognition by the cellular receptor [29]. Even though the number of the reactive amino groups is less than antibody molecules, the crystal structure of EGF (Figure 1a) indicates all the three amino residues are potentially reactive to COOH-terminated nanoparticles to yield three distinct orientations. Therefore, it is still important to use an EGF variant that allows for site-directed chemical coupling. One possible and commonly used approach is taking advantage of intrinsically lysine-free mouse EGF (Figure 1c), where the amide coupling reaction can be limited to the N-terminal amine. However, mouse EGF is known to induce immune responses in human, thereby this strategy is not suitable for the future medical applications. Moreover, the binding constant of mouse EGF has been reported to be 3-fold lower than human EGF [30]. Therefore, we took the genetic recombination approach to minimize the change in the sequence of human EGF.

Herein, we designed three EGF variants (RR, RS, and SR) without lysines in the EGF. These EGF variants can be site-selectively conjugated to the surface of GNP through the single primary amine at the N-terminus. In the soluble form, two EGF mutants (RS and SR) exhibited almost identical abilities to activate ERK to WT, but the remaining one (RR) showed weaker activities (Figure 3). It confirmed that the replacement of two lysine residues into RS and SR did not interfere intrinsic activity of EGF, except for the RR substitution. Upon conjugation of the two promising EGF mutants (RS and SR) plus WT to 15 nm GNPs at the surface density of ~8 molecule/NP, all of them retained the activity to induce ERK phosphorylation (Figure 7) and exhibited growth inhibitory effects (Figure 8). In our previous study [18], we have demonstrated the EGF-GNP-induced cellular death was mediated by the sustained activation of ERK. Therefore, the observed higher ERK phosphorylation as well as the growth inhibitory effects for the SR mutant over WT suggest the boosting of the same pathway by replacing WT EGF with the SR mutant. Among the two mutants, only the SR-GNPs showed significantly enhanced activities over WT-GNPs. On the other hand, the RS-GNPs only showed enhanced ERK phosphorylation within the lower nanoparticle concentrations (Figure 7b) although the EGF orientation has been optimized for both RS-GNPs and SR-GNPs. As one possibility, we hypothesized that the difference between the two mutants may be due to the binding affinity of them to EGFR inside cells. Based on this, we did the *in situ* binding assay of mutant as well as WT EGF to the intracellular target (EGFR), and found that the K_d_ value of SR is about twice as low as that of RS and WT (Figure 9). It is considered that the cytotoxic activities of EGF-GNPs are owing to the multivalent interactions between EGF-EGFR and the following EGFR condensation in the membrane rafts [16,18]. Therefore, slightly enhanced binding activity of SR-EGF toward EGFR might be amplified through the multivalent interactions, thereby leading to prolonged interactions between EGF-GNPs and EGFR and enhanced growth inhibitory effects. On the other hand, the number of EGF molecules conjugated to the GNP surface is 8 in the present study, which is smaller than those reported earlier [16,18]. Therefore, the entropic reward caused by the orientation control was not so effective in the RS mutant, resulting in the observed selective enhanced growth inhibition in the GNP conjugates of the SR mutant. Therefore, we do not exclude the possibility for the enhanced anti-cancer effects for the RS mutant by increasing the surface density of conjugated EGF on the nanoparticles. Such detailed analysis will be conducted in the forthcoming papers.

## 5. Conclusions

In this work, we developed lysine-free recombinant EGF mutants, where two intrinsic lysine residues were replaced with either serine (S) or arginine (R), to improve anti-cancer effects of EGF-GNPs conjugates by controlling the orientation of EGF against nanoparticle surface. Among the tested EGF mutants (RS, SR), the GNP conjugate of the SR mutant showed enhanced biological activities and growth inhibition in EGFR-over expressing skin cancer cell line A431. Through a series of biochemical analysis, thus observed activity enhancement of the SR is not solely attributed to the orientation control, but to increased binding activities of the mutant to EGFR. These results strongly validate the present strategy to control the modality of the EGF molecule at the nanoparticle surface in the development of more potent and efficient EGF-GNP conjugates, and further suggest the SR-GNPs as a candidate for cancer therapy.
